# Septic Arthritis of the Shoulder Following Ozone Therapy: A Case Report

**DOI:** 10.7759/cureus.101935

**Published:** 2026-01-20

**Authors:** Fidel Cortez, Eduardo Roncolato, Mariana Machado, Vitória Cubas

**Affiliations:** 1 Orthopedics and Traumatology, Mackenzie Evangelical University Hospital, Curitiba, BRA; 2 Medicine, Mackenzie Evangelical Medical School of Paraná, Curitiba, BRA

**Keywords:** case reports, efficacy, inflammation, ozone therapy, septic arthritis

## Abstract

Ozone therapy does not yet have a fully understood mechanism of action, and its use should be approached with caution. Septic arthritis, an acute joint infection, requires immediate intervention due to its severity and the risk of complications such as sepsis and permanent damage. This is a qualitative case report based on medical record analysis, along with a literature review on ozone therapy. A 65-year-old male patient with HIV, hypothyroidism, and systemic arterial hypertension presented with right shoulder pain, which worsened after undergoing ozone therapy. Upon admission to the emergency department, he exhibited signs of inflammation, restricted range of motion, a 60 cm³ subcutaneous fluid collection, and bone rarefaction on imaging. Surgical debridement was performed, with drainage of purulent secretion and collection of material for culture, which identified *Staphylococcus aureus*. Treatment with ciprofloxacin and sulfamethoxazole + trimethoprim was initiated for four weeks. The patient developed dry necrosis and necrosuppurative acute inflammation, with no signs of malignancy. After six weeks, laboratory improvement and pain resolution were noted. At six months, the patient reported good clinical progress and no symptoms. Although a temporal association was observed between ozone therapy and the onset of symptoms, this case report does not establish a causal relationship. Given the limited scientific evidence and regulatory concerns surrounding ozone therapy, its use should be approached with caution.

## Introduction

Ozone has demonstrated therapeutic effects in local applications, such as the treatment of external wounds. Its use in the form of a transcutaneous gaseous ozone bath has become the most practical and useful method [1]. However, its mechanism of action is not yet fully understood and must be applied with caution, as it can be harmful [2]. For this reason, the Brazilian Federal Council of Medicine considers it an experimental procedure [3], and the national regulatory agency Agência Nacional de Vigilância Sanitária (ANVISA) has approved ozone therapy only for dental and aesthetic purposes [4]. Inappropriate use may lead to adverse effects [5], as ozone dissolves in plasma and can generate oxidative stress through active metabolites released [6].

Septic arthritis is a joint infection characterized by acute inflammation and anatomical and functional deterioration [7]. It most commonly affects the knee and hip joints, although it can occur at any age [8], particularly in patients with comorbidities or immunosuppression [9]. Delayed treatment can result in bone loss and permanent joint dysfunction [8]. Clinical symptoms include joint pain, swelling, and fever (>38.5°C), with *Staphylococcus aureus* being the most common pathogen [10]. Complications include sepsis, permanent joint damage, amputation, osteomyelitis, and death, with sequelae such as chronic pain, limited mobility, and osteoarthritis [7].

This is a qualitative case report based on medical record analysis, approved by the Ethics Committee for Research of Faculdade Evangélica Mackenzie do Paraná affiliated with the Instituto Presbiteriano Mackenzie (approval number: 7.399.045), along with a literature review on ozone therapy. The study was conducted at the Mackenzie Evangelical University Hospital of Paraná, Curitiba, Brazil, and the patient provided informed consent.

## Case presentation

**Figure 1:**
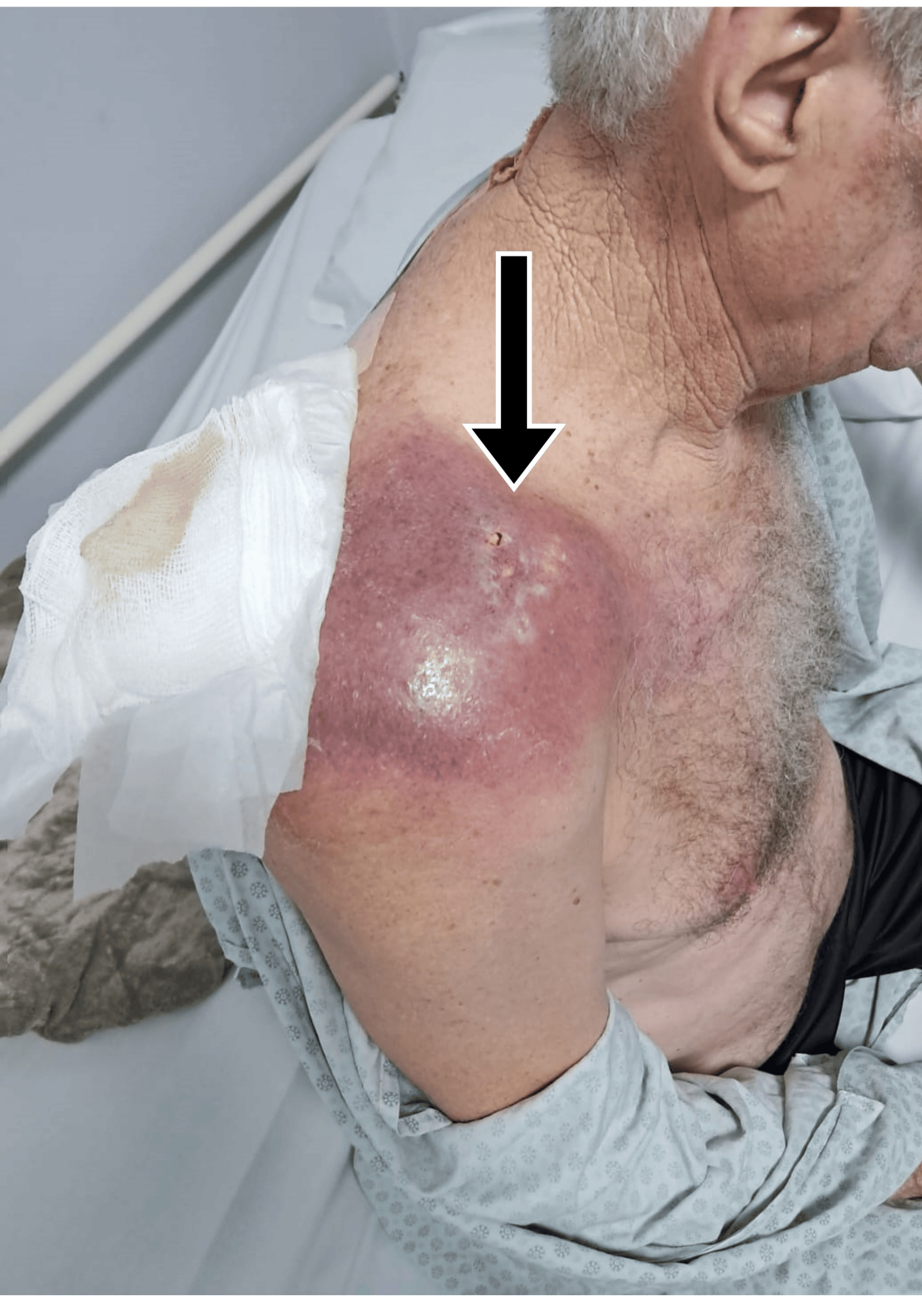
Inspection of soft tissues during hospital admission on September 8, 2024. Inflammatory signs are noted (arrow).

**Figure 2:**
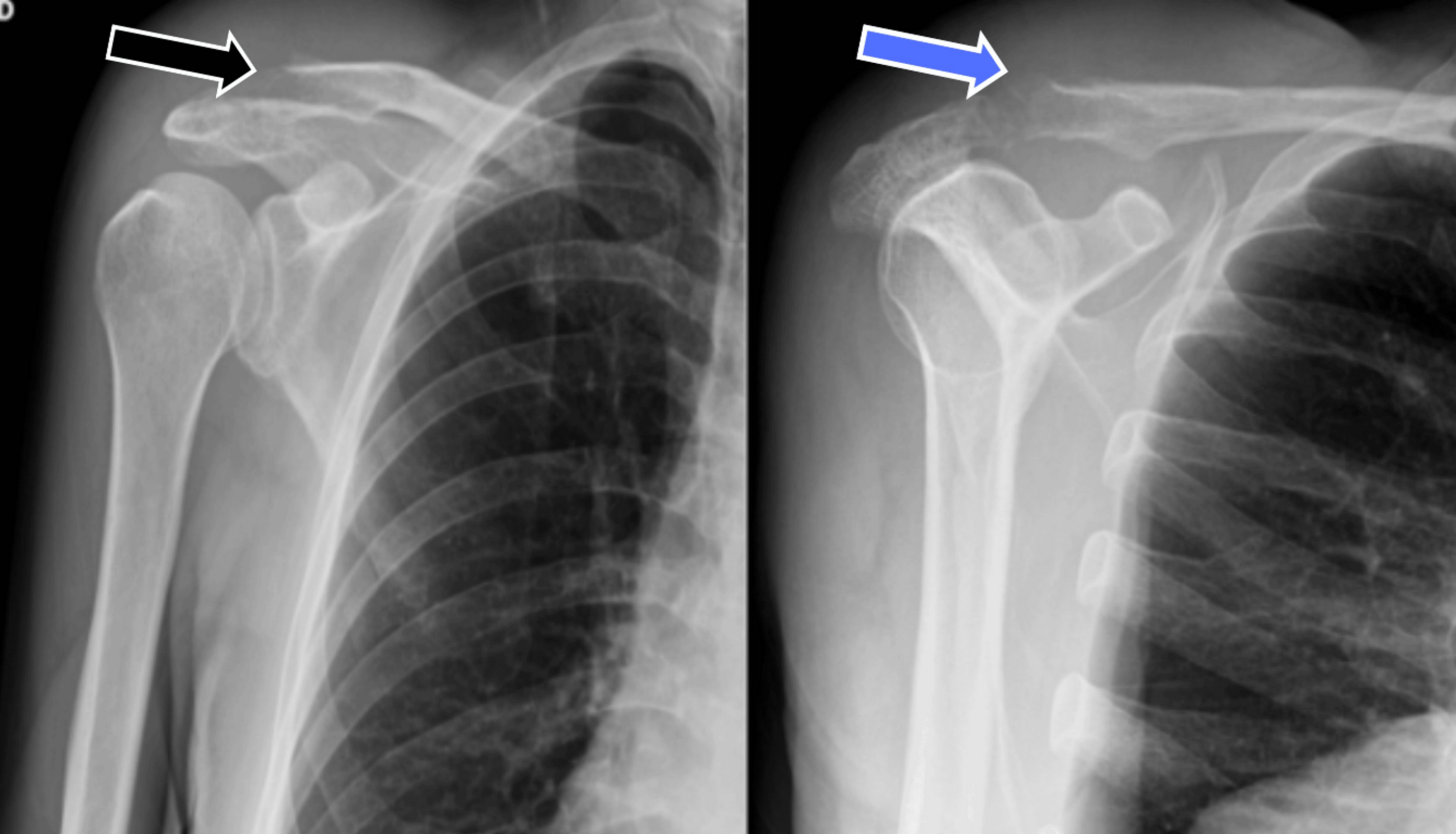
Anteroposterior (black arrow) and lateral (blue arrow) X-rays of the affected limb at the time of admission showing bone involvement (September 8,2024).

The patient, a 65-year-old male with hypothyroidism, systemic arterial hypertension, and HIV (with low viral load), was admitted to the emergency department on September 8, 2024, complaining of right shoulder pain lasting two years. He reported undergoing a single session of ozone therapy with a physical therapist 15 days prior in an attempt to relieve symptoms, which led to significant clinical worsening. Physical examination revealed inflammatory signs in the right shoulder (Figure 1), regional hyperemia, and impaired range of motion. An ultrasound performed the same day revealed a poorly defined 60 cm³ subcutaneous fluid collection. A right shoulder X-ray showed marked bone rarefaction in the acromial end of the clavicle and the acromion of the scapula, degenerative changes in the acromioclavicular joint, and increased soft tissue volume (Figure 2). 

Initial management included surgical debridement of the right shoulder on September 13, 2024, which revealed a large amount of purulent discharge, an infected bursa, necrotic tissue, and distal clavicular bone involvement. Samples were collected for culture and histopathological analysis. Three days later, the patient reported preserved muscle strength and sensitivity, with mild pain, and was discharged for outpatient oral antibiotic therapy (ciprofloxacin and sulfamethoxazole + trimethoprim) for four weeks. The choice of sulfamethoxazole + trimethoprim can be scientifically justified by its proven effectiveness against *Staphylococcus aureus* and demonstrated clinical efficacy in osteoarticular infections, and the possibility of prolonged use with an acceptable safety profile.

**Figure 3:**
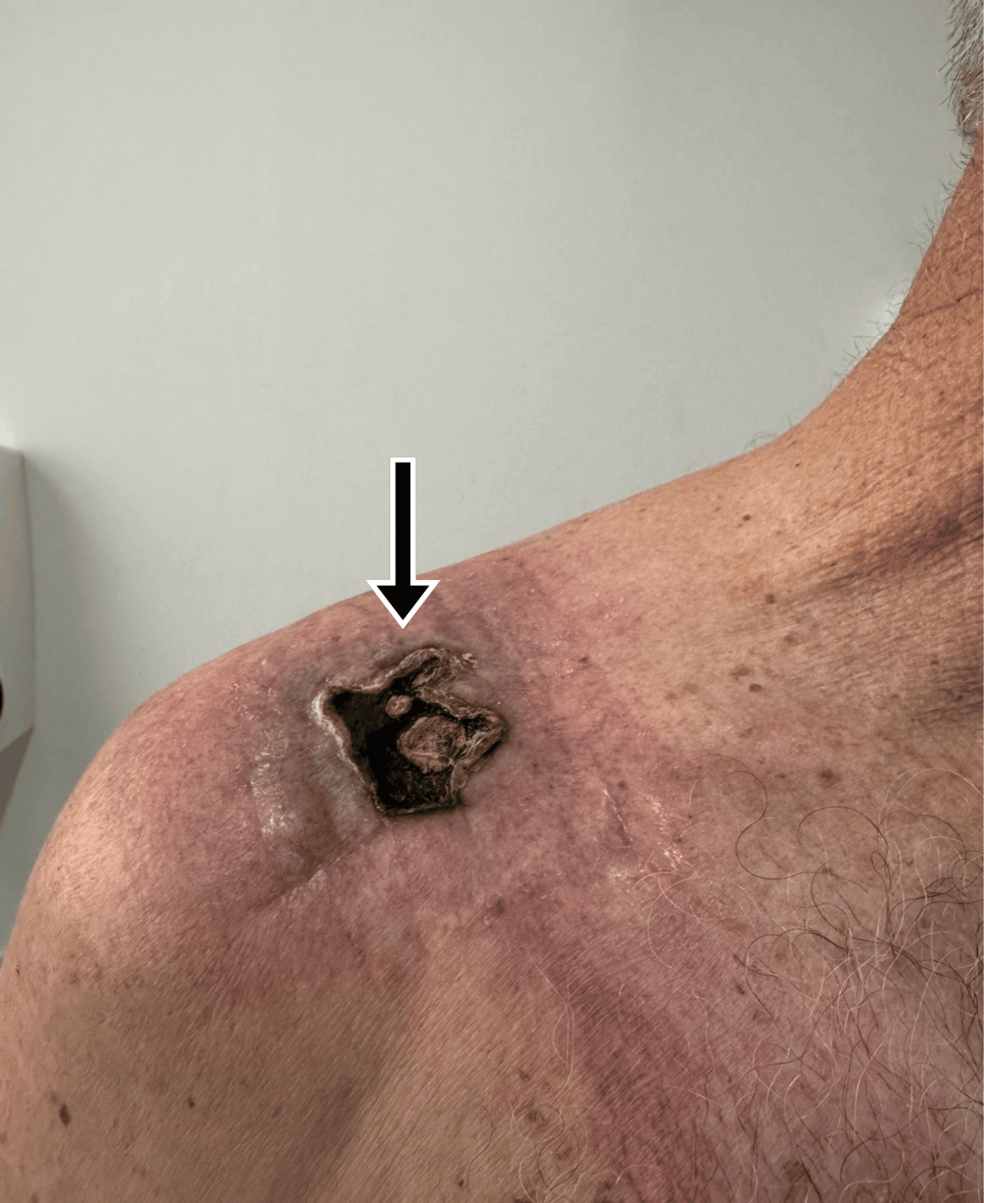
Inspection of soft tissues at admission, one week after surgical intervention. Dry necrosis in the anterior right shoulder area, purulent discharge at the wound edges, well-approximated sutures, and serous drainage upon pressure are noted (arrow; September 25, 2024).

Seven days post surgery, the patient returned for follow-up with dry necrosis in the anterior right shoulder area, purulent discharge at the wound edges, well-approximated sutures, and serous drainage upon pressure (Figure 3). Culture was positive for *Staphylococcus aureus*, and histopathological analysis revealed fibromuscular tissue with intense necrosuppurative acute inflammation, without malignancy.

**Figure 4:**
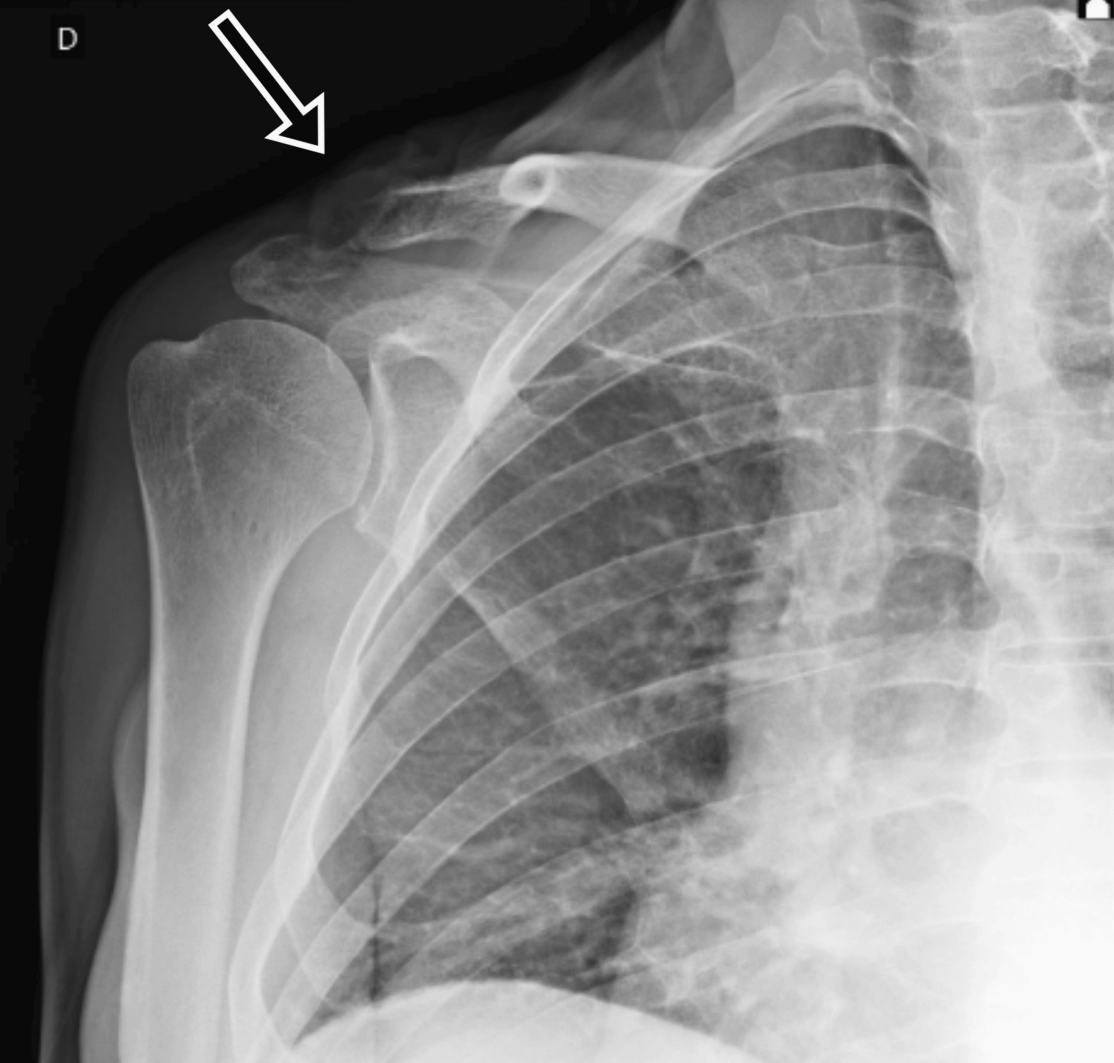
Anteroposterior radiograph of the affected limb; degenerative joint changes are noted (arrow; October 23, 2024)

**Figure 5:**
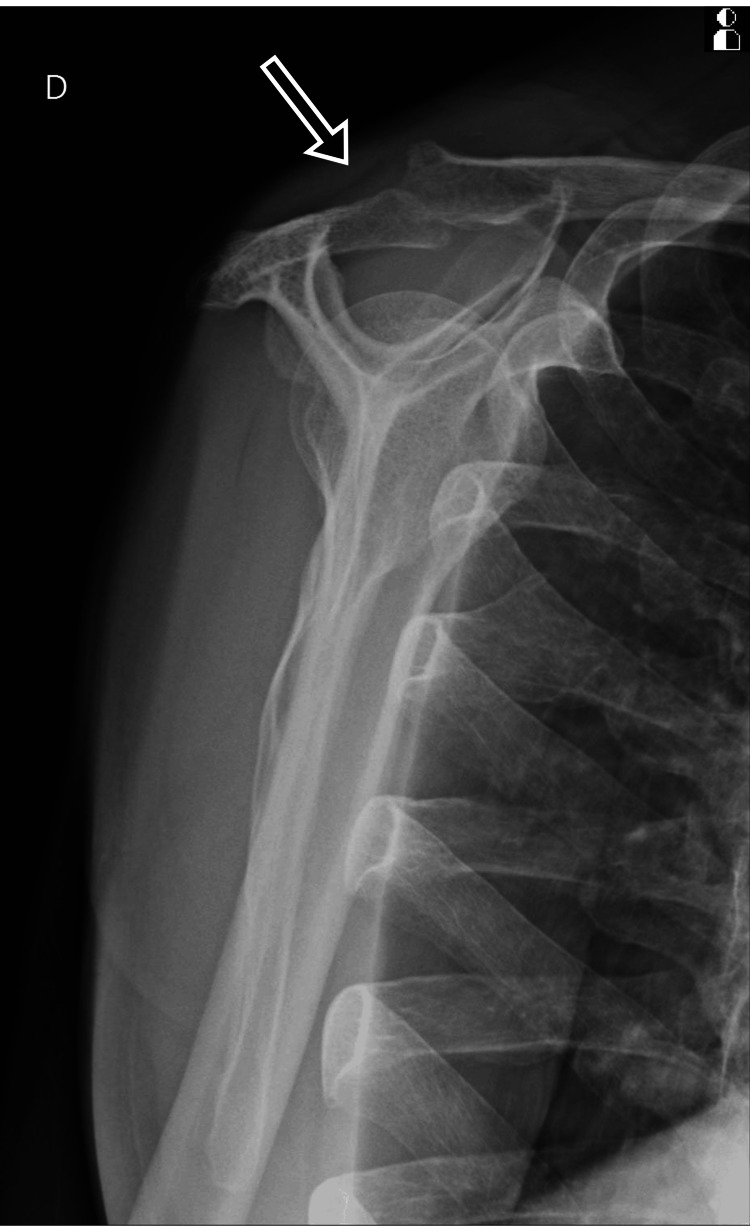
Lateral X-ray of the affected limb; degenerative joint changes are noted (arrow; October 23, 2024)

At the six-week follow-up (October 23, 2024), lab results indicated improving inflammatory markers (Table 1), and radiography showed degenerative joint changes (Figures 4, 5), though the patient reported no pain.

**Table 1:** Patient’s laboratory test progression WBC: white blood cells; CRP: C-reactive protein; ESR: erythrocyte sedimentation rate

Parameters	September 8, 2024	October 3, 2024	October 19, 2024	November 9, 2024
WBC (/mm³)	7.770	-	3.730	3.780
CRP (mg/dL)	27.74	3.48	5.99	1.72
ESR (mm/1st hour)	106	50	30	8

**Figure 6:**
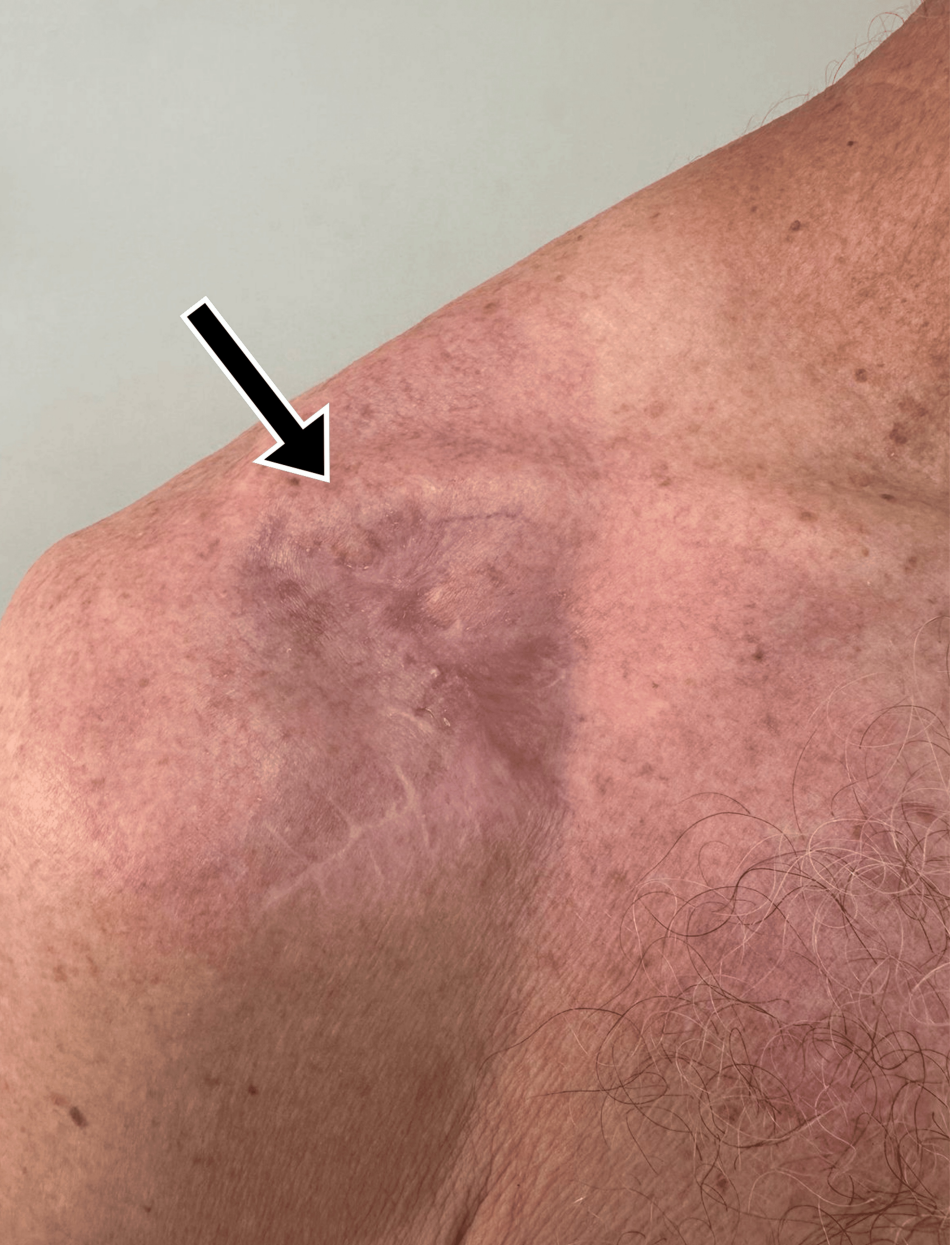
Inspection of soft tissues six months after surgical intervention; good recovery is noted (arrow; March 27, 2025)

At the final six-month follow-up, he reported being well, with no pain and good clinical recovery (Figure 6).

## Discussion

This case report illustrates how the growth of unauthorized medical practices, lacking scientific support and being used indiscriminately, can be highly detrimental to patient health. The Brazilian Federal Council of Medicine states there is insufficient evidence to confirm the efficacy of ozone therapy [3], and some of its side effects may result in cellular damage or even cell death [5]. In line with these concerns, this case demonstrates that improper use of ozone therapy may lead to severe and difficult-to-manage complications.

Although the patient had well-controlled HIV with a low viral load, HIV infection itself is a recognized risk factor for infectious complications. Therefore, while severe immunosuppression was not present, a degree of increased susceptibility to infection cannot be completely excluded.

Septic arthritis is a medical emergency with high morbidity, leading to sequelae, hospitalizations, invasive procedures, and mortality [7]. Contrary to the typical pattern described in the literature, where the knee and hip are more frequently affected [9], this case involved the shoulder joint.

Consistent with existing literature, the causative agent identified was *Staphylococcus aureus*, and treatment consisted of joint drainage and antibiotic therapy (ciprofloxacin and sulfamethoxazole + trimethoprim) for four weeks [9]. The delay between symptom onset and initiation of treatment is associated with orthopedic complications [9], which was evident in this patient, who sought care at an advanced stage, already exhibiting bone rarefaction and degenerative changes at hospital admission [10].

With appropriate treatment, the patient showed improvement in laboratory and radiographic findings, as well as symptom resolution and functional recovery. Common complications include spread to soft tissues and permanent joint damage [7], which aligns with the findings in this case.

It is important to emphasize that this case report does not establish a causal relationship between ozone therapy and septic arthritis. The association observed is temporal, based on clinical worsening following a single ozone therapy session. Other contributing factors, including patient-related comorbidities and procedural aspects, cannot be excluded. Nevertheless, this report highlights a potential risk associated with unregulated or improperly performed ozone therapy and underscores the importance of caution, regulation, and further high-quality studies to better evaluate its safety profile.

## Conclusions

Engaging in unverified practices without scientific support or regulatory approval poses a significant risk to public health. Septic arthritis, meanwhile, constitutes an orthopedic emergency that can result in irreversible damage, even after acute resolution. In this context, ozone therapy remains classified as an experimental procedure by the Brazilian Federal Council of Medicine. Thus, there is a pressing need for rigorous studies evaluating its efficacy, safety, adverse effects, and long-term consequences before its widespread use is considered.
